# Transcatheter aortic valve replacement for severe aortic regurgitation in quadricuspid aortic valve: a case report

**DOI:** 10.3389/fcvm.2025.1716669

**Published:** 2025-12-17

**Authors:** Abdullah Aljudaibi, Mark Sonbol, Haytham Allaham

**Affiliations:** University of Maryland Medical Center, Baltimore, MD, United States

**Keywords:** quadricuspid aortic valve, TAVR, aortic regurgitation, transcatheter aortic valve replacement, valve thrombosis, thromboembolic coronary events

## Abstract

A 49-year-old woman with a congenital quadricuspid aortic valve presented in cardiogenic shock due to severe aortic regurgitation. She underwent transfemoral transcatheter aortic valve replacement with a 29 mm self-expanding prosthesis. The procedure was technically successful with minimal paravalvular leak and significant symptomatic improvement. However, her long-term course was complicated by medication nonadherence, valve thrombosis, thromboembolic coronary events, and progressive heart failure. This case highlights the anatomical and technical challenges of transcatheter valve therapy in non-tricuspid anatomy, including annular sizing and valve anchoring without calcification, and underscores the critical importance of post-procedural anticoagulation adherence and meticulous follow-up to prevent thrombotic complications.

## Introduction

Quadricuspid aortic valve is a rare congenital malformation characterized by four aortic cusps instead of the usual three, affecting less than 0.05% of the population (1), often leading to progressive aortic regurgitation that becomes clinically significant in the fourth to sixth decades of life. Surgical aortic valve replacement or valve repair has been the standard intervention for symptomatic aortic regurgitation in quadricuspid aortic valve patients. However, many patients present with comorbidities that increase surgical risks. Transcatheter aortic valve replacement has emerged as a viable alternative for high-risk patients, yet the unique anatomical features of quadricuspid valves pose significant procedural challenges. Limited data exist on transcatheter interventions in this context, particularly for pure regurgitation without stenosis. This case addresses a clinical gap by illustrating the feasibility, challenges, and long-term considerations of transcatheter therapy in quadricuspid aortic valve-related regurgitation.

## Case presentation

A 49-year-old woman with a known history of quadricuspid aortic valve, ischemic cardiomyopathy, coronary artery disease with previous drug-eluting stents in the left anterior descending and right coronary arteries, hypertension, and chronic kidney disease presented in cardiogenic shock. On arrival, her blood pressure was 140/86 mmHg, mean arterial pressure 106 mmHg, pulse 90 bpm, respiratory rate 18 per minute, oxygen saturation 99% on room air, and afebrile. Physical examination revealed signs of poor perfusion and volume overload, including cold extremities, jugular venous distension, and peripheral edema. Laboratory findings indicated acute kidney injury, mildly elevated liver enzymes, and elevated lactic acid levels.

**Figure 1:**
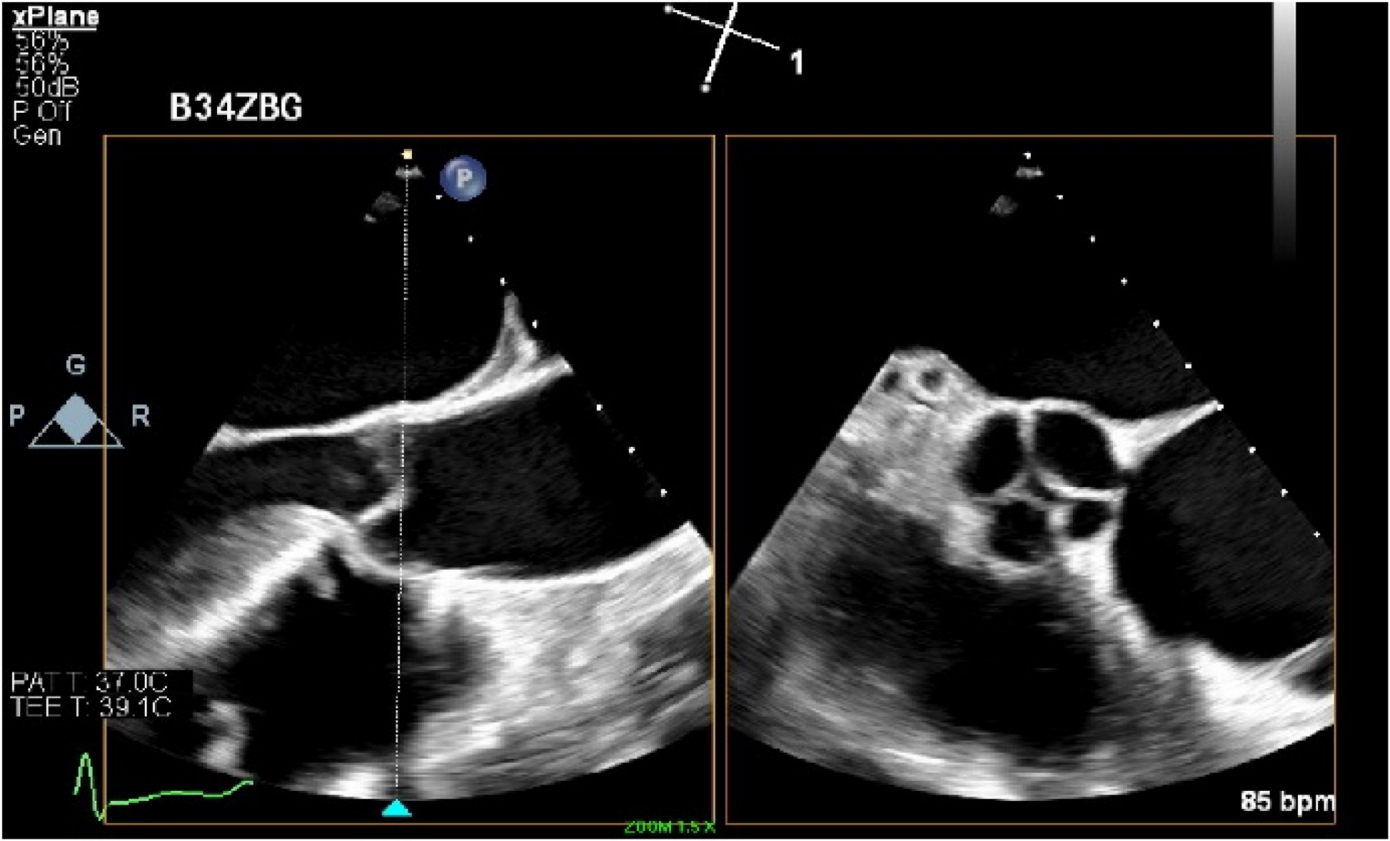
Image of the quadricuspid aortic valve captured via transesophageal echocardiography (TEE) in bi-plane view, highlighting the rare four-cusp morphology.

Initial transthoracic echocardiography revealed severe left ventricular dysfunction with an ejection fraction of 15%, a severely dilated left ventricle (end-systolic diameter 60 mm), and moderate to severe aortic regurgitation in the setting of a quadricuspid aortic valve without stenosis (Figure 1). Despite initiation of intravenous diuretics and inotropic support, her condition worsened, and she was transferred to our institution. Right heart catheterization demonstrated mildly elevated right-sided filling pressures, mild pulmonary hypertension, normal left-sided filling pressures, and a low cardiac index despite milrinone infusion. Left heart catheterization demonstrated patent coronary stents. A transesophageal echocardiogram (TEE) confirmed moderate to severe aortic regurgitation, normal aortic root dimensions, and moderate to severe mitral regurgitation. The native aortic valve demonstrated a quadricuspid morphology consistent with a Hurwitz and Roberts Type B configuration, characterized by three equal cusps and one smaller accessory cusp.

**Figure 2:**
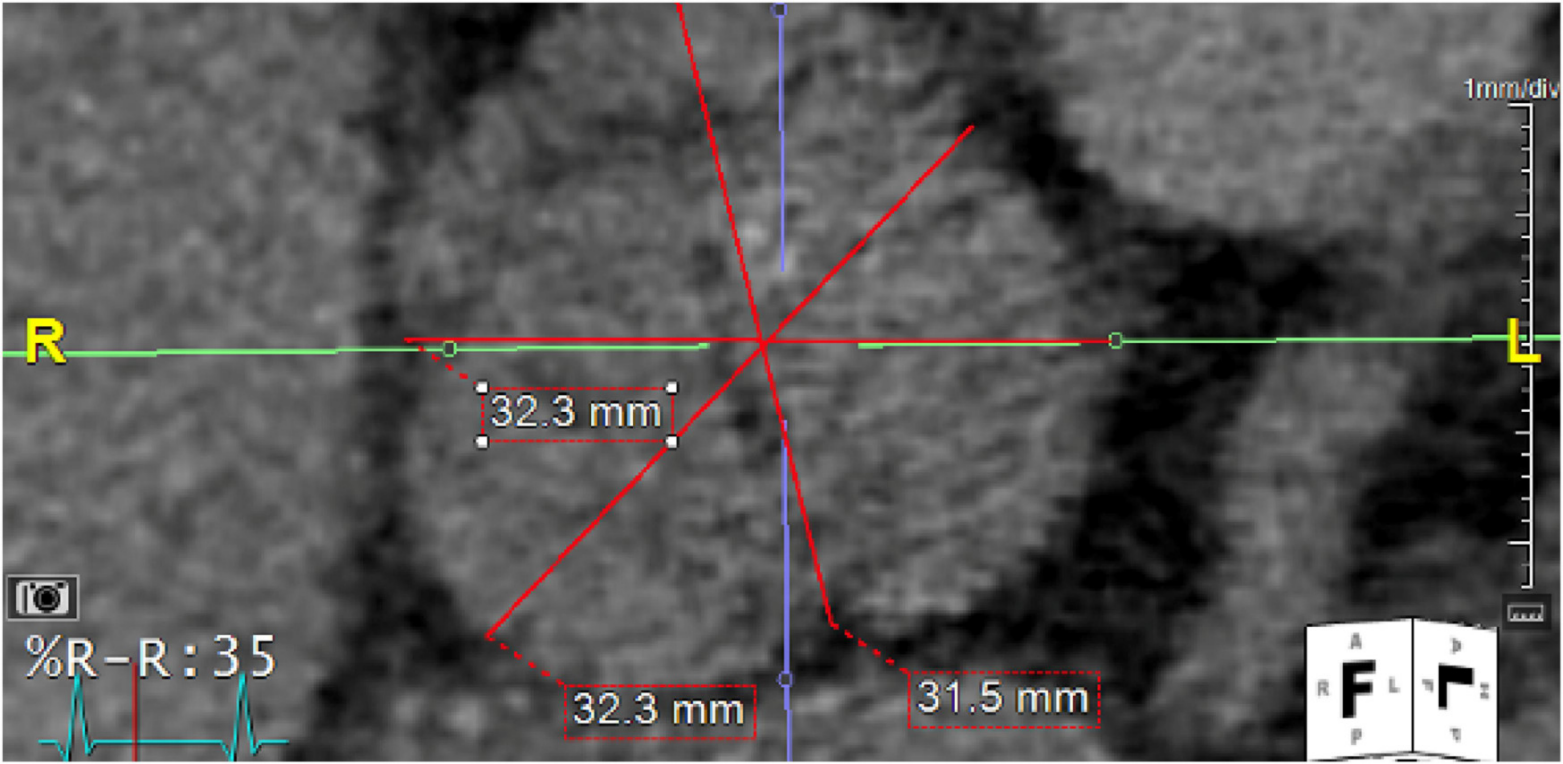
Computed tomography (CT) imaging illustrating the dimensions of the aortic annulus, critical for pre-procedural planning of valve sizing.

**Figure 3:**
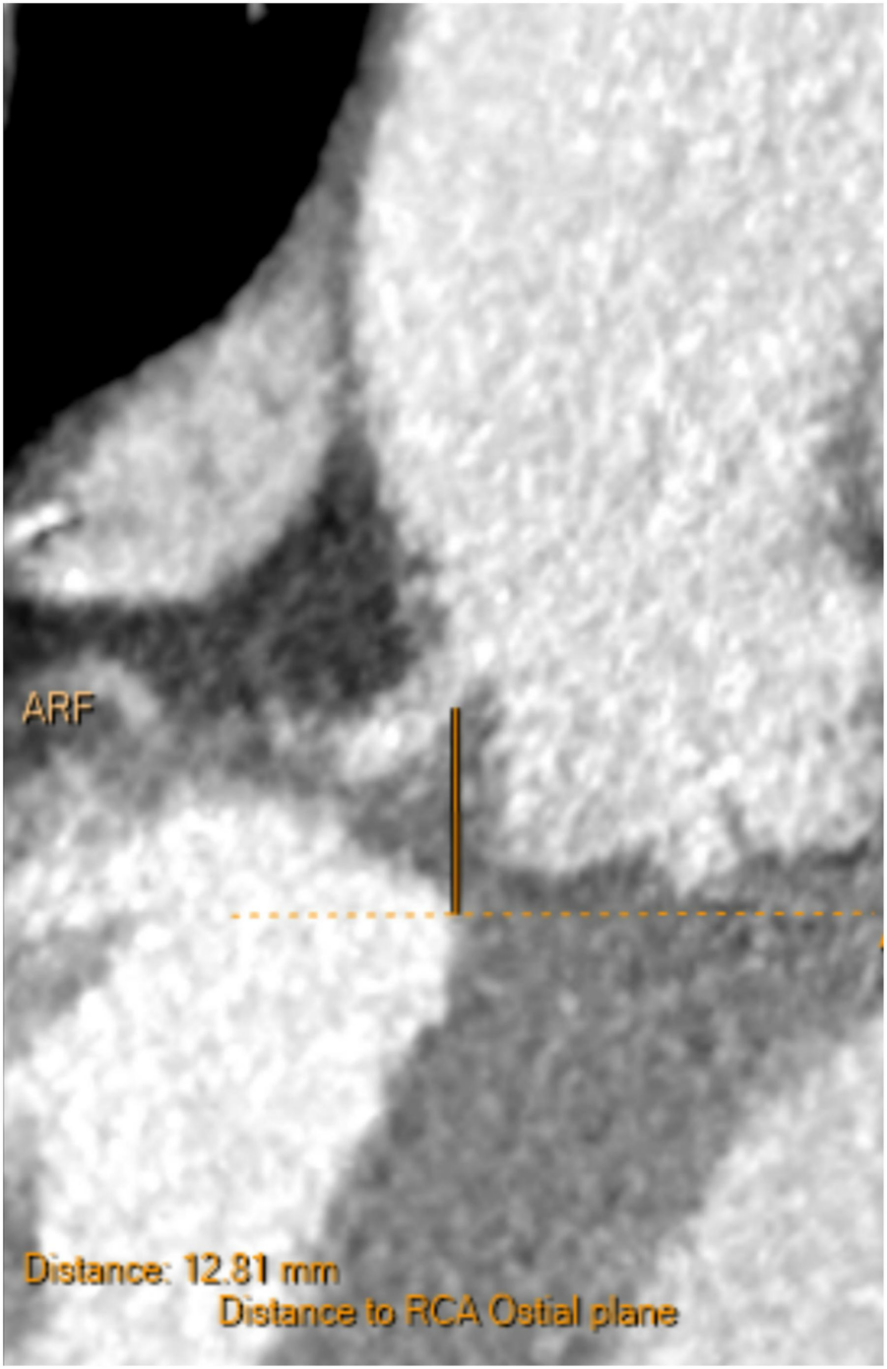
Pre-procedural CT showing the native right coronary ostium 12.8 mm above the annular plane, indicating a safe coronary height prior to valve implantation. No leaflet calcification or sinus sequestration was observed.

**Figure 4:**
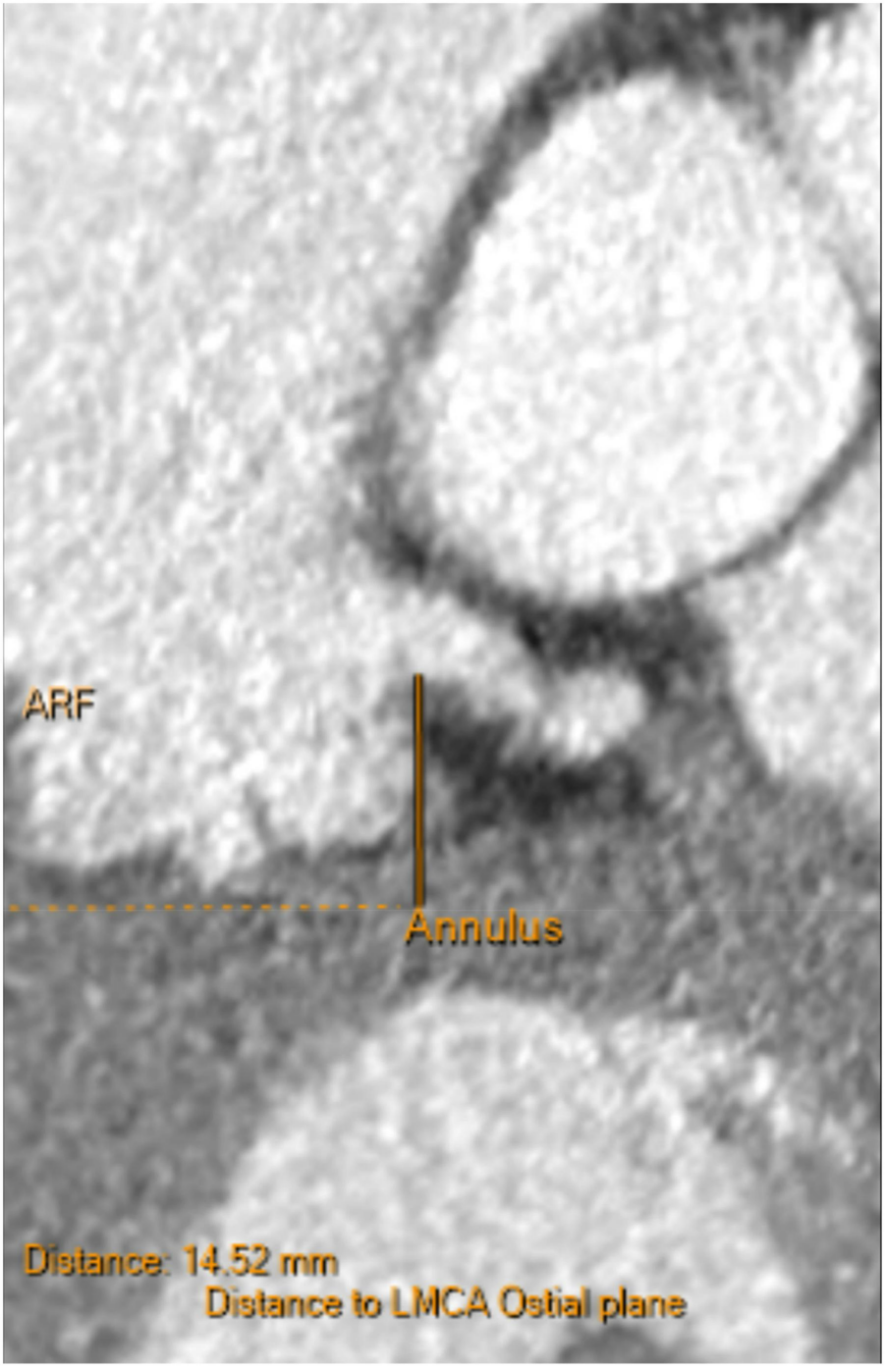
Pre-procedural CT demonstrating the left main coronary ostium 14.5 mm above the annular plane, confirming adequate clearance and favorable anatomy for TAVR. These measurements help exclude low coronary height as a mechanism for later ischemic events.

Given the patient's high surgical risk (Society of Thoracic Surgeons score of approximately 8% for combined aortic and mitral valve replacement), the multidisciplinary heart team recommended transcatheter aortic valve replacement (TAVR) to address the severe aortic regurgitation and potentially improve candidacy for advanced therapies like left ventricular assist device or transplant. Pre-procedural computed tomography demonstrated an annular perimeter of 71 mm (area 3.91 cm^2^) and absence of leaflet calcification (Figure 2). Coronary ostial heights measured 12.8 mm for the right coronary artery and 14.5 mm for the left main coronary artery (Figures 3, 4). Both were above the 10–12 mm thresholds associated with increased obstruction risk, indicating favorable anatomy for valve deployment. Primary access was via the right common femoral artery, prosthesis used was a 29 mm Medtronic CoreValve Evolut PRO+ self-expanding valve. Post-implant TEE confirmed appropriate valve positioning with trace paravalvular leak and preserved mitral valve motion. She was discharged on dual antiplatelet therapy with aspirin (81 mg daily) and clopidogrel (75 mg daily), with instructions for strict adherence.

One-week post-discharge, the patient was readmitted with chest pain and elevated troponin levels. Electrocardiogram was unchanged, but echocardiography showed persistent severe left ventricular dysfunction (ejection fraction 20%), mild paravalvular aortic regurgitation, and mean/peak aortic gradients of 2 mmHg and 3 mmHg, respectively. Notably, the left ventricular outflow tract velocity time integral (LVOT VTI) had decreased to 7 cm, from a postoperative value of 30 cm. Coronary angiography revealed a total occlusion of the mid-left anterior descending artery (LAD), proximal to the previously placed mid-LAD stent, with TIMI 0 flow beyond the occlusion. The lesion was initially managed with heparin, long inflation balloon angioplasty, and a single Integrilin bolus, but the vessel demonstrated persistent no-reflow and a diffusely thrombotic appearance. Aspiration thrombectomy using a Pronto device, followed by multiple intracoronary nitroprusside injections, restored TIMI 3 flow. An aortogram suggested thrombus on the aortic valve leaflets. Right heart catheterization confirmed cardiogenic shock, with markedly depressed cardiac indices, necessitating an intra-aortic balloon pump. Medical therapy included anticoagulation with heparin and inotropic support with dobutamine.

**Figure 5:**
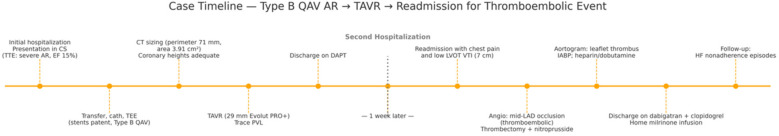
Clinical timeline showing two hospitalizations. The first admission involved TAVR for severe aortic regurgitation in a quadricuspid aortic valve. The second, one week later, was for thromboembolic occlusion of the mid–left anterior descending artery and prosthetic leaflet thrombosis.

Review of follow-up records indicated early non-adherence to dual antiplatelet therapy (DAPT) after discharge, which likely contributed to prosthetic valve thrombosis and downstream coronary embolization. She was ultimately discharged on dabigatran (150 mg twice daily) and clopidogrel (75 mg daily), alongside a home milrinone infusion. Follow-up over several months revealed ongoing heart failure challenges due to medication nonadherence (Figure 5). Her functional status remained limited, with no return to baseline daily activities.

## Discussion

Quadricuspid aortic valve (QAV) is a rare congenital anomaly and is characterized by four aortic valve cusps instead of the normal three cusps (1). The Hurwitz and Roberts classification categorizes QAV into six subtypes (A–F) based on cusp size. Type A (four equal cusps) and type B (three equal cusps and one smaller cusp) are the most common (2). These structural anomalies result in irregular annulus, presenting technical challenges during valve interventions. Nonetheless, despite these difficulties, TAVR has achieved remarkable success rates (3).

A systematic review published in 2025 of 31 cases reported successful transvalvular aortic deployment in all patients with minimal complications (3). A similar study by Liu et al. reported five QAV patients treated with TAVR had minimal residual AR and good device function. In addition, all patients had noticeable symptom improvement within a month, with most progressing to NYHA class II or better by a year (4). TAVR for pure AR is technically challenging, due to the risk of valve embolization, given the lack of leaflet calcification, which typically provides an anchor for the valve prosthesis. To mitigate the risk of embolization, pre-procedural planning is essential. This includes precise annular measurements to guide significant valve oversizing (typically >20% compared to 10% in aortic stenosis). Also, deeper deployment (e.g., 70:30 aorto-to-LVOT ratio, compared to the typical 80:20 ratio) can help in aortic valve anchoring. Because of this complexity, advanced imaging like 3D CT and intraoperative TEE can play a key role in guiding accurate valve placement and minimizing complications during the procedure (5).

Balloon-expandable valves (e.g., SAPIEN 3) provide excellent valve sealing, but they carry a higher risk of valve embolization in non-calcified anatomy (4). On the other hand, self-expanding valves (e.g., CoreValve, Venus-A) may better anchor in non-calcified annuli with a lower risk of embolization (6). Notably, a newer generation of devices for pure AR, such as the JenaValve and J-Valve, is emerging as promising options in less calcified aortic cusps (4). Complications of TAVR in QAV include coronary obstruction, paravalvular leaks (PVL), and conduction issues (3% requiring a pacemaker) (3). In rare cases, valve-in-valve procedures may be necessary to address PVL in some patients with QAV (6). To date, no strokes or annular ruptures have been reported in the QAV TAVR experience.

**Figure 6:**
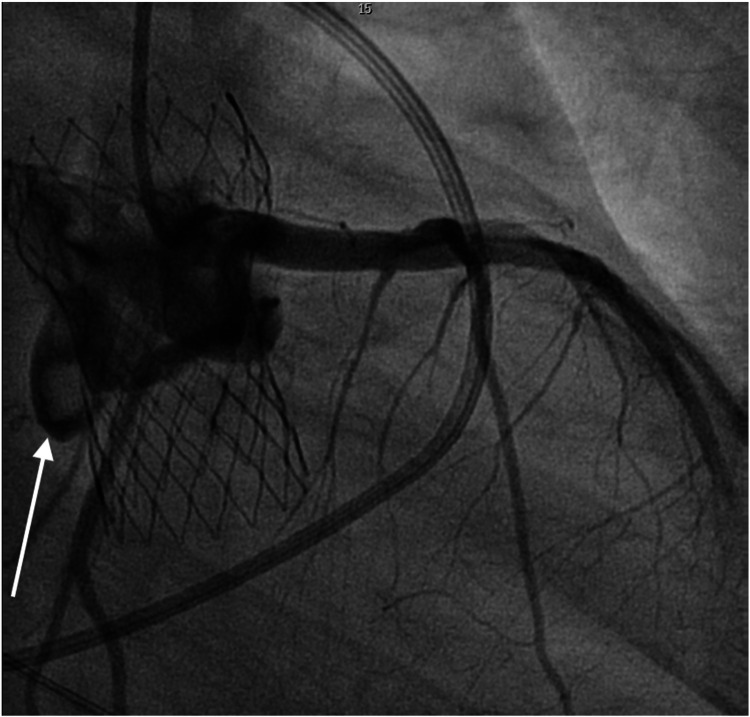
Aortography during the second hospitalization demonstrating mobile filling defects along the prosthetic valve leaflets (white arrow), consistent with leaflet-level thrombus.

Prosthetic leaflet thrombosis was strongly suspected in this case, supported by both imaging and hemodynamic findings. Aortography during readmission demonstrated filling defects localized to the prosthetic valve leaflets, consistent with thrombus formation (Figure 6). The valve position remained stable, with no increase in paravalvular leak or structural distortion, further favoring a functional obstruction from thrombus. Although leaflet thrombosis is uncommon with the Medtronic CoreValve/Evolut system, its occurrence in this case is best attributed to early post-discharge interruption of dual antiplatelet therapy, which is a well-recognized precipitant. Other procedural or anatomic contributors are less clear, as existing data do not firmly link valve size, oversizing, or non-calcified anatomy to thrombosis risk. This highlights that even in technically uncomplicated implants, pharmacologic factors may dominate the early thrombotic milieu.

**Figure 7:**
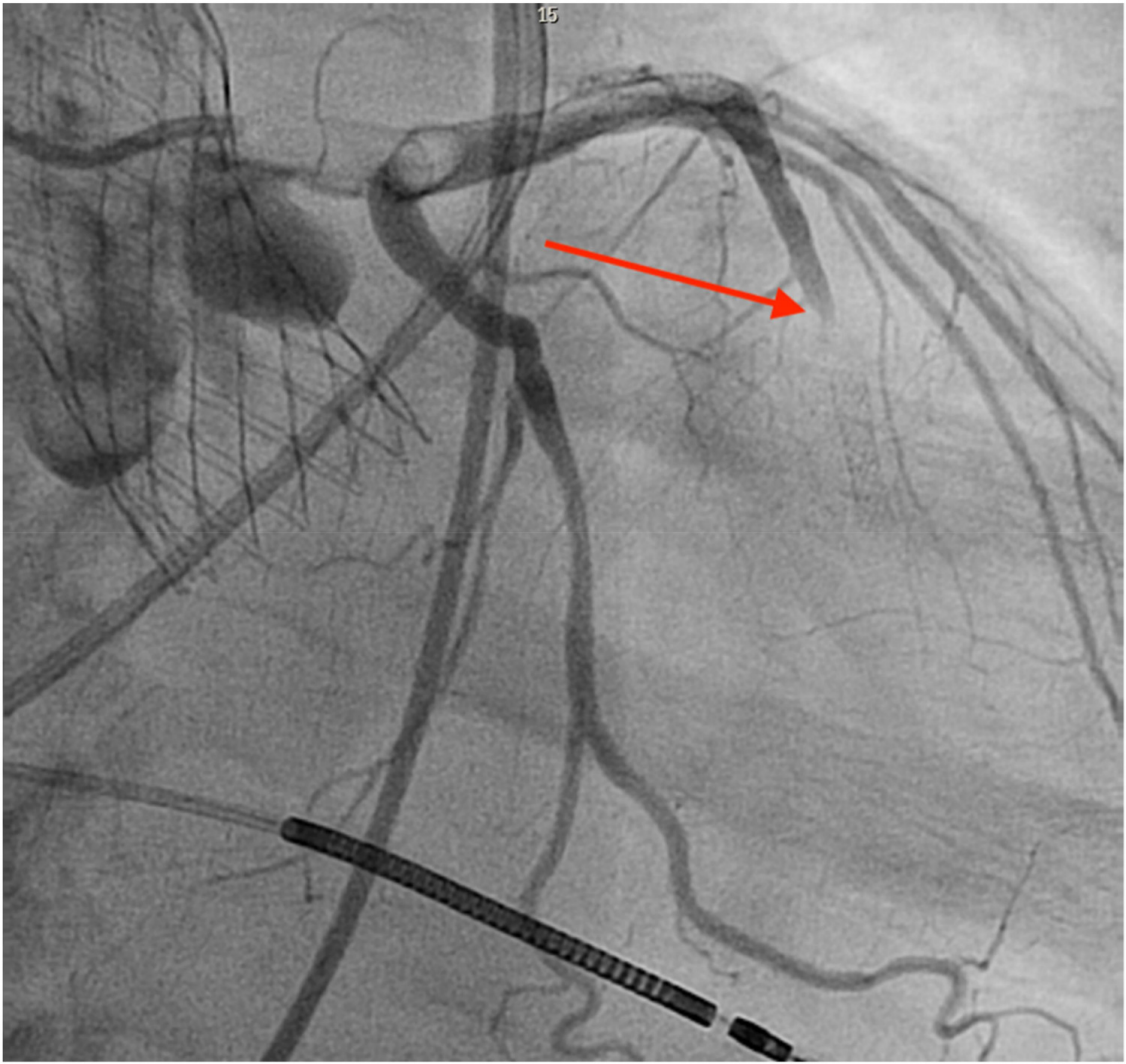
Coronary angiography showing a total occlusion of the mid LAD (TIMI 0 flow) with thrombotic material filling the lumen (red arrow). The segment proximal to the prior stent appears smooth and without focal plaque rupture, suggesting an embolically mediated occlusion.

In this context, the leaflet thrombus provided a plausible source for distal embolization, prompting careful evaluation for associated coronary involvement. The coronary vessel segment appeared otherwise smooth and free of underlying atherosclerotic irregularity (Figure 7), and the previous stent was widely patent on angiography performed shortly before TAVR, making atherosclerotic plaque rupture unlikely. The appearance was therefore most consistent with a thromboembolic process, potentially originating from the prosthetic valve. Following initiation of systemic anticoagulation with dabigatran, the patient demonstrated clinical stabilization, supporting a thrombotic etiology. This case underscores that even self-expanding valves with a low reported incidence of thrombosis can develop leaflet thrombus under adverse hemodynamic and pharmacologic circumstances, particularly in non-calcified anatomy.

Our case highlights the complication of post-procedural valve thrombosis and coronary thromboembolism, precipitated by early discontinuation of dual antiplatelet therapy after discharge.

## Conclusion

TAVR is a less invasive alternative to surgery for high-risk patients with QAV and severe AR. Success requires meticulous pre-procedural imaging, the appropriate choice of device, and expert procedural skills. As technology and experience evolve, TAVR outcomes in QAV continue to improve. Continued case reporting is key for TAVR techniques and guiding device development effectively (7).

## Data Availability

The original contributions presented in the study are included in the article/Supplementary Material, further inquiries can be directed to the corresponding authors.

## Supplementary material

Supplementary Video 1 Color Doppler imaging across the quadricuspid aortic valve, demonstrating moderate to severe aortic regurgitation prior to intervention.

Supplementary Video 2 Aortic angiography performed post-TAVR, showing no paravalvular leak (PVL) or aortic insufficiency (AI) with well-seated valve positioning.

Supplementary Video 3 Post-TAVR transthoracic echocardiography (TTE) in parasternal long-axis view, confirming normal valve function and effective deployment.
